# Subcortical Band Heterotopia Presented With Refractory Epilepsy and Reversible Aphasia

**DOI:** 10.7759/cureus.16990

**Published:** 2021-08-08

**Authors:** Reaz Mahmud

**Affiliations:** 1 Neurology, Dhaka Medical College, Dhaka, BGD

**Keywords:** seizure, subcortical band heterotopia, dcx gene, aphasia, refractory epilepsy

## Abstract

Subcortical band heterotopia (SBH) is a rare neurodevelopmental disorder due to mutation in the DCX or LIS1 gene. It is predominantly a disease of females. Its presentation varied widely, ranging from mild epilepsy and mental retardation to refractory epilepsy and severe mental retardation. Here, a case of a 22-year-old lady with refractory seizure is reported. She also had expressive aphasia which had reversed after adjustment of the anti-epileptic drugs and control of the seizure. Her MRI of the brain revealed a band of complete gray matter deep to the pachygyric cortex and an electroencephalogram (EEG) revealed bi-frontal slow waves.

## Introduction

Subcortical heterotopia is a rare neurodevelopmental disorder of the human brain. There are three subtypes of subcortical heterotopia: (a) nodular, (b) laminar, and (c) subcortical band heterotopia (SBH) [1-2]. The presence of bilaterally symmetrical, heterotopic gray matter in between the ventricles and the cortex characterizes SBH [3]. It is a classic example of deficient neuronal migration associated malformations [4].

The predominant cause of SBH is the mutations in the DCX or LIS1 gene [5]. The mutated genes produce proteins with altered structure or function, resulting in impaired interaction which is needed for neuronal migration [6]. The improper neuronal migration causes misplacement of the neuron, leading to the formation of a band beneath the cerebral cortex [7-8].

Here, a female patient who was admitted to the Neurology Department, Dhaka Medical College with poorly controlled seizures and acquired expressive aphasia is presented. After the appropriate investigations, the final diagnosis of SBH has been made. Informed consent was obtained from the patient’s mother (approved by Ethical Research Committee, ERC) as the patient was not communicable at presentation. Ethical permission was obtained from the ethical review committee of Dhaka Medical College (ERC-DMC/ECC/2021/76).

## Case presentation

**Figure 1:**
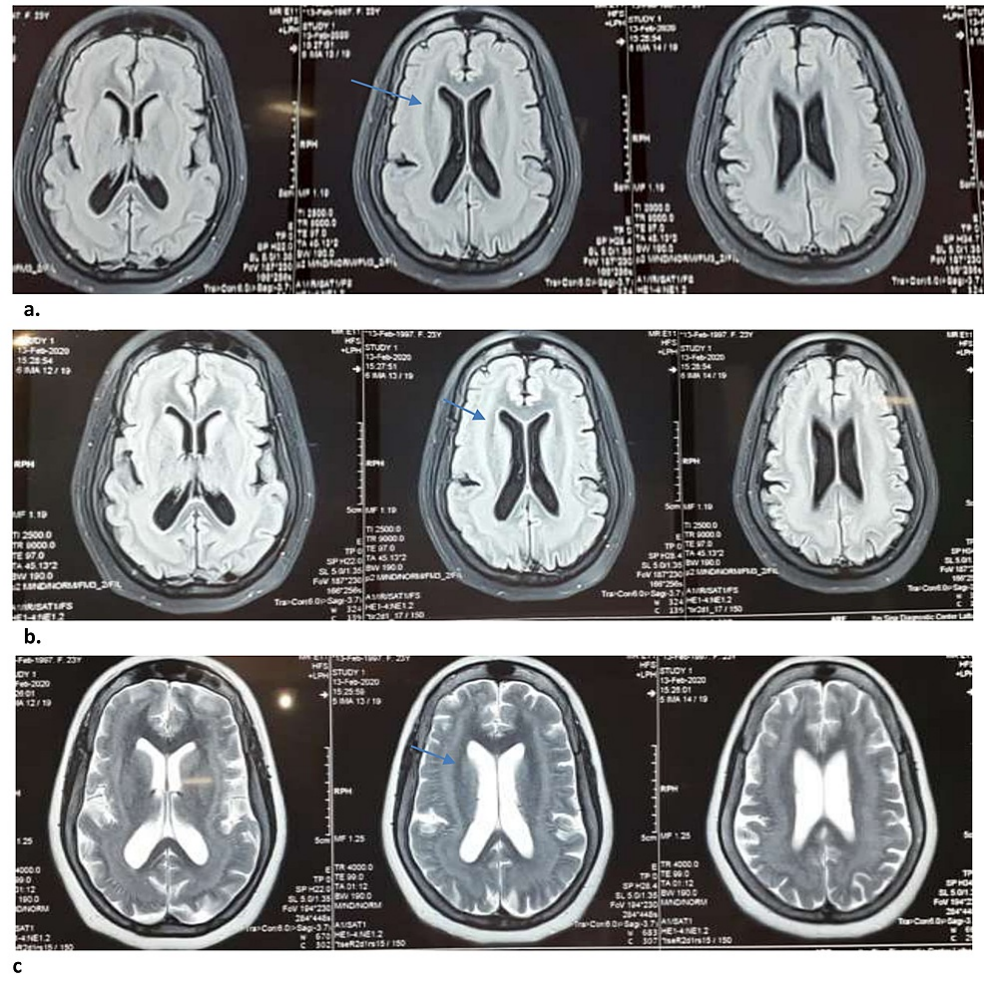
MRI of Brain T1 (top row, a ) T2 (middle row, b) FLAIR (bottom row, c) sequence revealed a complete band of gray matter located deep to, and roughly paralleling, pachygyric overlying the cortex. The thickness of the band was marked in posterior parietal and occipital region. FLAIR, fluid-attenuated inversion recovery

A 22-year-old, unmarried lady presented with recurrent seizures since her six years of age. The seizure started in her right hand and then became generalized. The seizure persisted for two to three minutes. There was no preceding prodrome, aura, or automatism. There was post ictal confusion but no post ictal paralysis. Her milestones of development were normal up to the age of six, except that she had mild mental retardation. She did not go to school. She could speak normally prior to her onset of the seizure. She had developed progressive speech regression since her 10 years of age and had become mute at her 12 years. She could follow the simple command. There was no family history of epilepsy. Her seizure was not controlled even after taking several anticonvulsants. Since her 10 years of age, she has experienced almost daily seizures. At the time of presentation, she experienced 5-10 seizures per day. She also had occasional rage attacks. She was on sodium valproate 1000 mg/day, carbamazepine 800 mg/day, and levetiracetam 1000 mg per day which were started at her 6, 9, and 16 years of age respectively. The patient was apathetic. She had expressive aphasia. There was no focal weakness. Superficial and deep tendon reflexes were normal. Her MRI of the brain (Figure 1) revealed a complete band of gray matter located deep and parallel to pachygyric overlying cortex.

**Figure 2:**
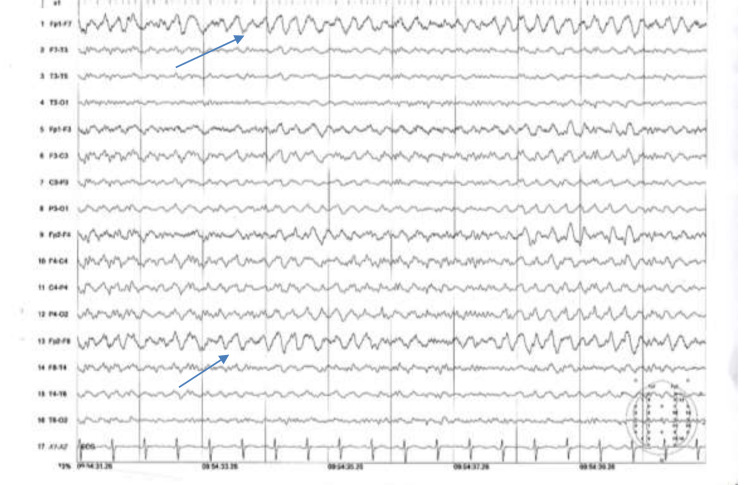
EEG in bipolar montage revealed bi-frontal intermittent slow wave mainly in stage 1 sleep. EEG, electroencephalogram

The thickness of the band was more in the posterior part of parietal and occipital cortex. An electroencephalogram (EEG) (Figure 2) revealed bi-frontal slow waves which were marked during stage 1 sleep.

We gradually discontinued carbamazepine over 15 days, as we thought that it could exaggerate the seizure. We increased the doses of sodium valproate and added clobazam. We added clobazam 10 mg daily and increased the dose of sodium valproate to 1500 mg daily. At this, her seizure frequency was gradually decreased and her speech had gradually improved. After 15 days of discontinuing carbamazepine, her seizure was fully controlled. She began to speak and communicate during her 30 days of follow-up. Consequently, follow-up after six months revealed that she had experienced two transient brief attacks of seizure during the course of the period. Detailed history revealed that the seizures occurred during the missed dosage of the drugs. So, we did not make any dose adjustment and advised her strictly not to miss the dose of anticonvulsants further. Her final drugs were sodium valproate 1500 mg, levetiracetam 1000 mg, and clobazam 10 mg per day.

## Discussion

Subcortical band heterotopia was defined by Dobyns et al. [9] as a neurodevelopmental disorder that consists of “bilateral and symmetric ribbons of gray matter located in the centrum semiovale between the cortex and ventricular walls, which are separated from both by layers of white matter.” It is found that most individuals with SBH have DCX or LIS1 gene mutations [4]. DCX gene mutation is an X-linked dominant disorder. So SBH shows a striking skewing of sex ratio to females. LlS1 gene is located on 17p13.3 [5]. As DCX is carried on the X chromosome of males, mutations in DCX will usually have classical Lissencephaly in males whereas females have SBH [4-5]. The onset of the seizure may occur at any age, predominantly in the first decade but occasionally delayed until the second or third decade [3]. The reported patient was a female who had developed seizure at her six years of age. Patients with SBH will usually have mild-to-moderate intellectual disability and a wide range of seizures [9]. The severity of intellectual disability also varies and depends on the thickness of the heterotopic band [3-4, 9].

The seizure types are highly variable from patient to patient and vary from focal, complex partial to generalized seizures [9]. Simple/complex partial seizures (~70%) are mostly prominent, followed by drop attack (~30%), absence seizure (~25%), and myoclonic seizure (~ 15%). Generalized tonic-clonic seizure either primary or secondary may occur in 20%-50% of the cases [6, 8-9]. Patients with West syndrome and Lennox-Gastaut syndrome have also been described [7]. Importantly, a high proportion of drug resistance (65%-78%) is reported [6, 8-9]. Those with more severe MRI abnormalities have significantly earlier seizure onset and are more likely to develop Lennox-Gastaut syndrome [10].

In these instances, the patient is presented with mild mental retardation with poorly controlled seizures. Thereafter she also developed speech regression. So our initial diagnosis was Landau-Kleffner syndrome (LKS). The point against the diagnosis of LKS was that it is more common in males [11]. In LKS the aphasia is mainly verbal auditory agnosia and the loss of receptive language is followed by expressive aphasia [11]. The carbamazepine was thought to be the factor for worsening her seizure frequency. Carbamazepine was found to exaggerate different syndromic epilepsy of childhood including LKS [12-13]. So it was decided to gradually withdraw carbamazepine, increase the dose of sodium valproate, and add clobazam. To rule out any secondary etiology, an MRI of the brain with contrast was advised. The MRI of the brain of the patient revealed typical features of SBH. 

In the case of SBH MRI, it shows the characteristic appearance of symmetrically and circumferentially arranged smoothly marginated layer of gray matter beneath the cortex. It is separated from the overlying cortex and under­lying ventricle by layers of white matter. They do not contain blood vessels or cerebrospinal fluid (CSF) [9, 14]. The thicker the band of heterotopic neurons; the worse the disability and increased prevalence of developmental delay [7, 14]. The MRI of the patient (Figure 1) showed a typical appearance with a relatively thin band.

There is no specific pattern of EEG findings described in the current literature. Some authors found interictal anterior theta activity or intermittent rhythmic delta activity [15]. Others describe focal or multifocal spike and wave in the cetro temporal region [15]. Her EEG (Figure 2) revealed bifrontal rhythmic slow wave of delta range. Her genetic testing could not be done due to the unavailability of the testing facilities. After the adjustment of the dose of the drugs, her seizure was fully controlled and her speech was regained. So there was a potential seizure exaggeration by carbamazepine and might be speech regression due to uncontrolled seizure activity. The underlying mechanism, by which carbamazepine exacerbates certain seizure types and not others, remains unexplained. In some seizures, especially in the absence seizure the underlying mechanism is thought to be due to its influence on the thalamocortical oscillatory network [16]. Carbamazepine should be carefully given to patients with idiopathic generalized epilepsy, particularly those with typical absence seizures, juvenile myoclonic epilepsy, Lennox-Gastaut syndrome, LKS, and myoclonic epilepsies of childhood [16].

## Conclusions

In dealing with a patient with epilepsy syndrome or epileptic encephalopathy, an MRI of the brain should be mandatory. Adding and adjustment of the antiepileptic drug should also be rational. We have to be careful of adding carbamazepine in this type of patient. In the case of resistant epilepsy, potential seizure exaggeration by anticonvulsant drugs should be reviewed critically.
